# Mechanical Properties of Inflamed Appendix Tissues

**DOI:** 10.3390/biomedicines12112588

**Published:** 2024-11-12

**Authors:** Piotr Deptuła, Dawid Łysik, Przemysław Wolak, Grzegorz Król, Paulina Paprocka, Piotr Bijak, Dominika Ziembicka, Joanna Mystkowska, Robert Bucki

**Affiliations:** 1 Independent Laboratory of Nanomedicine, Medical University of Bialystok, PL-15222 Białystok, Poland; piotr.deptula@umb.edu.pl; 2 Institute of Biomedical Engineering, Bialystok University of Technology, PL-15351 Białystok, Poland; d.lysik@pb.edu.pl (D.Ł.); j.mystkowska@pb.edu.pl (J.M.); 3 Faculty of Medicine, Collegium Medicum, Jan Kochanowski University, PL-25369 Kielce, Poland; przemyslaw.wolak@ujk.edu.pl; 4 Department of Pediatric Surgery, Urology and Traumatology, Provincial Hospital in Kielce, PL-25736 Kielce, Poland; 5 Department of Microbiology and Immunology, Institute of Medical Science, Collegium Medicum, Jan Kochanowski University in Kielce, PL-25317 Kielce, Poland; grzegorz.krol@ujk.edu.pl (G.K.); paulina.paprocka@ujk.edu.pl (P.P.); rikssonia@gmail.com (P.B.); 6 Department of Public Health, Medical University of Bialystok, PL-15089 Białystok, Poland; dominika.ziembicka@umb.edu.pl; 7 Department of Medical Microbiology and Nanobiomedical Engineering, Medical University of Bialystok, PL-15222 Białystok, Poland

**Keywords:** atomic force microscopy, tissue rheology, mechanomarkers, mechanobiology, acute appendicitis

## Abstract

**Background/Objectives**: Histopathological examination enables visualization of morphological changes in cells and tissues. In recent years, there has been increasing interest in assessing the mechanical properties of tissues that cannot be determined by standard histopathological examinations. Mechanobiology is crucial in human physiology and holds promise for uncovering new diagnostic markers for disease processes such as carcinogenesis and inflammation. In this study, we concentrated on measuring the mechanical properties of appendix biopsy specimens to identify potential mechanomarkers of inflammation. Appendix tissues provided the opportunity to measure mechanical properties both with an atomic force microscope and a shear rheometer. **Methods**: The atomic force microscope AFM—NanoWizard 4 BioScience JPK/Bruker was used for the evaluation of the elastic modulus (i.e., Young’s modulus) of appendix tissues. Young’s modulus was derived from the Hertz-Sneddon model applied to force-indentation curves. The rheological properties of macroscopic samples were measured on a parallel-plate, strain-controlled shear rheometer Anton Paar MCR302. **Results**: The data collected suggest that elasticity, expressed as Young’s modulus and the storage modulus, could be considered a marker indicating appendix tissue inflammation. Young’s modulus of inflamed appendix tissues was found to be significantly lower than that of healthy ones, with an average reduction of 67%. Furthermore, it was observed that inflamed appendix tissues, in comparison to healthy ones, respond differently under varying axial and shear stresses, enabling their identification. **Conclusions**: Our findings suggest that the specific mechanical properties of inflamed vermiform appendices could serve as novel mechanomarkers for the early detection and monitoring of appendicitis.

## 1. Introduction

An important investigation in the diagnostic process of diseases is histopathological examination, which, using microscopic techniques, enables the visualization and analysis of morphological changes and the determination of markers of pathological changes based on immunohistochemistry, immunofluorescence, or RNA in situ colored markers in tissue sections [1,2,3,4]. Tissues can also be characterized by material features that are invisible in histopathological examination—mechanical (rheological) properties, which can be very helpful in the diagnosis of diseases [5,6,7].

Rheological properties are important for maintaining the proper functioning of organs. Recent studies have indicated that dysfunction of physiological processes during infection, inflammation and carcinogenesis generates structural changes in cells and/or the extracellular matrix (network consisting of macromolecules that surround, support, and give structure to cells and tissues), which cause changes in the rheological properties of cells and entire tissue structures [8,9,10,11,12,13,14,15,16,17,18].

It should be emphasized that mechanical changes may be observed earlier than those visible in histopathological examinations, and many factors determining the rheology of cells and tissues related to the health of the body cannot currently be assessed during routine histological assessment. In recent years, there have been reports that inflammation promotes cancer. For example, it has been proven that gastric infection caused by the bacterium *Helicobacter pylori* is usually associated with asymptomatic gastritis, and in the long term, it may lead to more serious clinical consequences, such as gastric ulcer, duodenal ulcer and gastric cancer [19,20,21]. Changes in the mechanical properties of inflamed tissues are less known compared to the mechanical properties of cancer tissues [6,17]. The ability to differentiate between the processes of inflammation and carcinogenesis is essential for developing specific tumor mechanomarkers and for exploring the transition from inflammation to cancer, which is crucial for early diagnosis. Undoubtedly, research and analysis of the mechanical parameters of tissues and organs are important to fully understand pathophysiological phenomena [8,14,22]. Mechanobiology plays a large role in human physiological processes. Moreover, it may have great practical potential from the perspective of learning new diagnostic methods for disease processes [14,15,23,24,25]. Finding new disease markers is essential for developing diagnostic methods that will accelerate and improve diagnosis, enhancing comfort for both doctors and patients.

The mechanics of biological samples are examined using tensiometers, atomic force microscopy (AFM), or shear rheometers, which allow for the determination of the rheological properties (visco-elastic properties for materials that exhibit both viscous and elastic characteristics when undergoing deformation) of living cells, biofilms, biomaterials or fresh tissue samples [10,12,14,15,22,26,27,28,29,30,31]. In recent years, attempts have been made to apply AFM in routine inflammation diagnosis, like gastric inflammations caused by the presence of *Helicobacter pylori* [17], pancreatic islet inflammation [32] or leukoplakia of human oral mucosa [25]. There have also been attempts to use these methods in routine cancer diagnosis, like colon cancer [14], oral carcinoma [25], gliomas [15], and breast cancer [33].

Mechanical measurements of cells and tissues in physiological and pathological states help determine how forces and changes in mechanical properties contribute to disease development and progression. Unfortunately, there is currently no standard procedure for these measurements. Clearly, more rheological studies using healthy and pathological tissue samples are still needed to relate ex vivo observations to in vivo mechanical processes.

The aim of this study was to assess the potential of using an atomic force microscope and a shear rheometer to evaluate the mechanical properties of inflamed tissues obtained from appendiceal biopsies. The study sought to identify potential mechanomarkers of inflammation that could aid in developing new methods for diagnosis, prevention, and treatment of this condition, as well as in differentiating it from carcinogenesis.

## 2. Materials and Methods

### 2.1. Tissue Samples

In this study, the series of tissues collected from children undergoing surgical removal of the appendix, clinically diagnosed as acute appendicitis at the Department of Pediatric Surgery, Urology and Traumatology, Provincial Hospital in Kielce, Poland, were examined. Tissue samples were obtained from both male and female patients at the age of 13–18 years old with confirmed inflammatory processes based on physical examination of the patient (palpation), ultrasound imaging, blood counts (including WBC white blood cell levels) and CRP protein levels. Due to the nature and timing of the surgical operations, the samples were frozen after excision in a special medium designed to minimize tissue structure damage during freezing (70% (DMEM + 10% FBS) + 20% FBS + 10% DMSO + tablet of cOmplete™, EDTA-free Protease Inhibitor Cocktail, F. Hoffmann-La Roche AG, Basel, Switzerland); 1 tablet per 50 mL extraction solution). The tissue samples for AFM and rheological testing were excised from the middle section of the appendices. AFM experiments were made maximally 2 h after sample thawing, and tissues were stored and kept in a culture medium during the experiment at room temperature. Before examination, the tube-shaped appendices were cut lengthwise, and the inner surface (the lumen of the appendix) was prepared for analysis. Due to the difficulty in obtaining three separate samples from completely healthy appendices, one appendix, which was excised occasionally from a patient with ovarian inflammation, was used as the control sample. Measurements were taken from three separate fragments excised from a single healthy appendix. In the case of inflamed tissue samples—for purulent inflammation and gangrenous inflammation, one fragment excised from each of three separate appendices was measured. Each control and inflamed sample were tested only once. Tissue samples were collected in accordance with an IRB protocol (11/2022) approved by the Bioethics Committee of the Collegium Medicum, Jan Kochanowski University of Kielce, Kielce, Poland.

### 2.2. AFM Measurements

**Figure 1:**
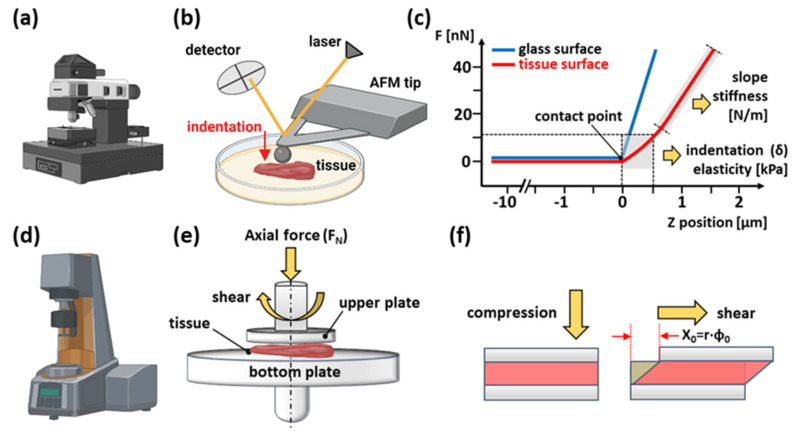
Schematic representation of two experimental settings. Rheological properties of vermiform appendix samples were tested using atomic force microscopy (**a**) and a shear rheometer (**d**). (**b**) Measurement visualization of Young’s modulus using AFM. The main AFM components are a cantilever with a spherical tip, a laser source, a photosensitive photodiode and a piezoelectric scanner for applying a normal force to soft tissue samples. (**c**) The compressive force applied as a function of a sample’s position in the Z-direction induces so-called force vs. distance curves. The difference between the AFM cantilever deflection on a stiff surface (blue curve) and the soft tissue (red curve) describes the deformation of the tissue under the load applied. This enables the determination of the sample modulus of elasticity (Young’s modulus). (**e**) Rheological experimental setup—the upper sandblasted 8 mm diameter parallel plate system. (**f**) Shear forces, in combination with sample compression, were applied by rotating the upper plate in a direction parallel to the sample. The deflection of the measuring plate by the angle *φ* was converted into shear deformation (figure was created with Biorender.com).

In this study, an AFM and a shear rheometer were used to measure the mechanical properties of appendix biopsy specimens. Small millimeter-scale tissue samples were measured with a NanoWizard 4 BioScience JPK Instruments Bruker AFM (Bruker Nano GmbH, Berlin, Germany) working in the Force Spectroscopy mode. Force indentation curves were collected using a silicon nitride cantilever with a nominal spring constant of 0.1 N/m and spring constant measured in the range of 0.09–0.1 N/m using the thermal tune method, with a 5.0 μm spherical tip (Figure 1c). The cantilevers were manufactured by NanoAndMore (NanoAndMore GmbH, Wetzlar, Germany).

Tissue samples were glued onto a 35 mm Petri dish and immersed in DMEM for measurements at room temperature. During the experiment, the bead-tissue contact area ranged from 12 to 35 μm^2^, depending on the depth of spherical tip indentation. To account for cantilever bending, force curves were first taken from the rigid substrate and then from the compliant soft tissue sample. Up to 15 indentation maps consisting of 8 × 8 points corresponding to a scan area of 10 × 10 µm were made for each tissue sample. Indentation maps were taken from multiple places on the sample surface. The differences between the cantilever deflection on a stiff surface and the compliant sample define tissue deformation under the AFM tip load (Figure 1c). The elastic modulus (i.e., Young’s modulus) was determined based on the so-called force-versus-indentation curves obtained by plotting the force of deformation against the depth of indentation. Force-versus-indentation curves were fitted to the Hertz contact model using formulas described in the study [14]. The data/statistical analysis was carried out using a dedicated software—JPK Data Processing (Version spm-6.0.58).

### 2.3. Rheological Characteristics

The bulk rheological properties of tissue samples were characterized using a strain-controlled Anton Paar MCR302 rheometer (Anton Paar GmbH, Graz, Austria) equipped with parallel plate geometry (Figure 1). Tissues were carefully excised and shaped into discs with an 8 mm diameter steel punch for analysis. These disc-shaped samples were then placed on P600 sandpaper (DEXTER, Ronchin, France) affixed to the lower plate to prevent slippage during measurements. The upper plate, also 8 mm in diameter, had a sandblasted surface to further enhance grip and minimize potential movement artifacts. A Teflon sample hood (Anton Paar GmbH, Graz, Austria) was used to prevent heat loss and water evaporation from the tissues. The initial gap was set after the initial force of 0.1 N stabilized for approximately 60 s, which was taken as the point of contact with the sample. The measurements were carried out using the forced oscillation method, where deflections of the upper plate by a corresponding angle φ translated into a sinusoidal course of shear strain in the sample. By measuring the stress required to deform the sample, both the in-phase storage modulus G’ and the out-of-phase loss modulus G” were determined. Uniaxial deformation (tissue compression) was controlled by changing the distance between the parallel plates in the range of 0–40% of the initial sample height, with a 10% increment. The rheological testing protocol consisted of two tests: (1) oscillating shear strain with a frequency f = 1 Hz and an amplitude γ = 1% for 60 s at each compression level (ε = 0, 10, 20, 30, 40%) applied by the step-wise decrease in the gap height between the plates and (2) shear strain amplitude sweeps of γ = 0.1–100% with a frequency f = 1 Hz of the uncompressed (ε = 0%) and compressed (ε = 40%) tissue samples.

### 2.4. Statistical Analysis

**Figure 2:**
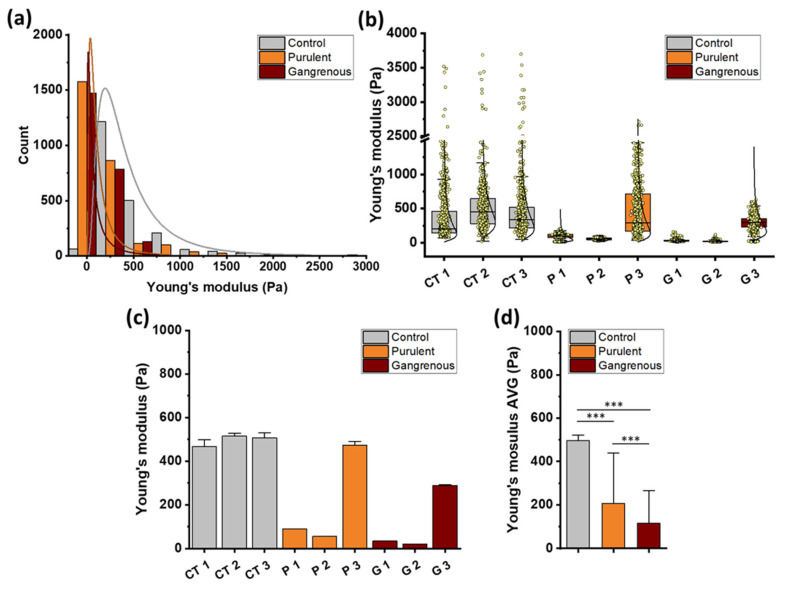
The Young’s modulus values obtained for control (CT), inflamed—purulent (P) and gangrenous (G) vermiform appendix using AFM indentation technique: (**a**) Young’s modulus distribution for control and inflamed tissues for all patients with a fitted probability density function of the log-normal distribution. (**b**) Young’s modulus distribution for each control (CT1–CT3), purulent (P1–P3) and gangrenous tissues (G1–G3) with a fitted probability density function of the log-normal distribution. (**c**) The mean values of tissues Young’s modulus ± standard deviation for each control, purulent and gangrenous tissue obtained from all force vs. distance curves. (**d**) The mean values (AVG) of tissues’ Young’s modulus ± standard deviation for each group of control and inflamed tissues. C1–C3—tissue samples excised from a single healthy appendix; P1–P3—tissue samples excised from three separate inflamed (purulent) appendices; G1–G3—tissue samples excised from three separate inflamed (gangrenous) appendices. Each sample was tested once. *** *p* ≤ 0.001.

**Figure 3:**
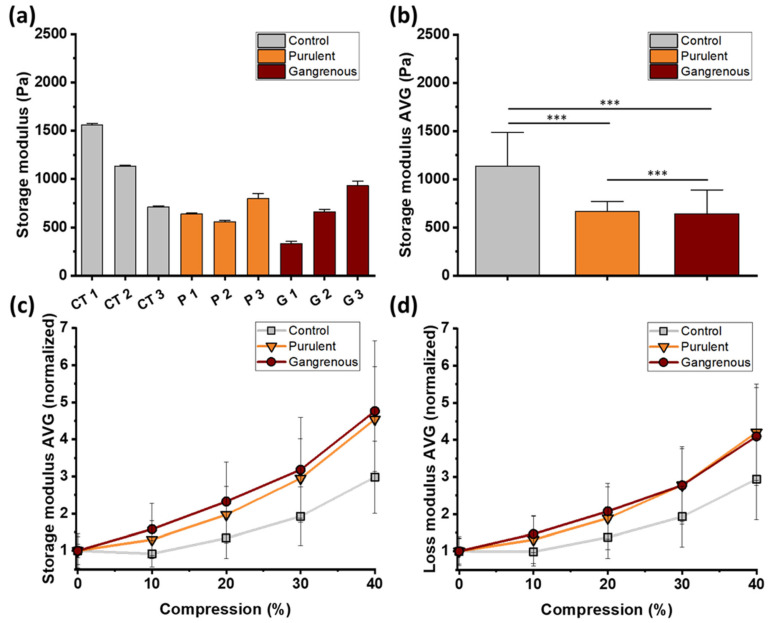
Rheological properties of the control and inflamed vermiform appendix: (**a**) Storage modulus values (G’) for each control (CT1–CT3), purulent (P1–P3) and gangrenous tissues (G1–G3). (**b**) The mean values of tissues’ storage modulus ± standard deviation for each group of control and inflamed tissues demonstrated smaller elasticity of inflamed tissues compared to control ones. (**c**) The normalized (AVG) storage modulus of control and inflamed tissues as a function of axial strain (compression up to 40% of initial tissue height). The compression stiffening effect is visible for both control and inflamed tissues, but inflamed tissues stiffen to a greater extent. (**d**) The normalized (AVG) loss modulus values (G’’) of tissues are expressed as a function of axial strain. Inflamed tissue in compression reacts by increasing elasticity more prominently compared to healthy tissue. Inflamed tissues become more elastic at impressing compression. C1–C3—tissue samples excised from a single healthy appendix; P1-P3—tissue samples excised from three separate inflamed (purulent) appendices; G1–G3—tissue samples excised from three separate inflamed (gangrenous) appendices. Each sample was tested once. *** *p* ≤ 0.001.

The significance of differences was determined using the two-tailed Student’s *t*-test. Statistical analyses were performed using OriginPro 9.65 (OriginLab Corporation, Northampton, MA, USA). *p* < 0.05 was considered to be statistically significant. In AFM measurements, average values of Young’s modulus are presented as mean ± SD, where the mean is the average value for each tissue from all force curves, and SD is a standard deviation (Figure 2c) or where the mean is the average value for three tissues belonging to a certain state (control, inflamed P, and inflamed G), and SD is a standard deviation (Figure 2d). In rheological characteristics, average values of elastic modulus G’ are presented as mean ± SD, where the mean is the average value for each tissue, and SD is a standard deviation (Figure 3a) or where the mean is the average G’ value for three tissues belonging to a certain state (control, inflamed P, and inflamed G), and SD is a standard deviation (Figure 3b).

## 3. Results

### 3.1. AFM Measurements

In this study, the rheological properties of inflamed appendix tissues were measured based on the example of appendix biopsy specimens obtained in the examination. An AFM microscope was used as a nano-indenter to measure micro-scale samples and a shear rheometer to measure macroscopic samples to determine the mechanical characteristics of the tissues. In the first experiment, the stiffness of inflamed and control appendix tissues was measured using an AFM microscope. These tests consisted of a series of cycles of loading and unloading the tissue surface with a constant force of 1 nN using a probe with a spherical tip (Figure 1).

The force-distance curves obtained were used to determine the Young’s modulus of tissue samples. This module was calculated using the dedicated JPK Data Pressing software. When analyzing the obtained data, attention was paid to the mechanical features that distinguish control tissues from inflamed appendix tissues, which can be used as mechanical markers of inflammation. Figure 2 shows the values of Young’s modulus obtained using the AFM technique.

Relative values of Young’s modulus distributions of control tissue samples (CT1–CT3), tissue samples with diagnosed purulent inflammation (P1-P3), and tissue samples with diagnosed gangrenous inflammation (G1–G3) are presented. Panel a shows an aggregate histogram of Young’s modulus values for all control and all inflamed tissues with the fitted log-normal probability density function. Panel b shows the distributions of Young’s modulus values measured for individual control and inflamed tissues with the fitted log-normal probability density function. Panel c shows the mean Young’s modulus values for individual control tissues, tissues with purulent inflammation, and tissues with gangrenous inflammation. Panel d shows the mean values ± standard deviation of the three tissue types examined. The aggregate histogram presented in Figure 2 shows the overall results obtained for all control and inflamed tissues examined in the study. The visible distributions of Young’s modulus values of inflamed appendix tissues are shifted towards lower values of elastic modulus. A clear difference can be observed between the stiffness of control and inflamed tissues. Young’s modulus values observed for control tissues are characterized by a broader distribution and are shifted towards higher values, while the distribution of Young’s modulus of pathological appendix tissues is characterized by sharper peaks in softer zones. A similar trend exists for the distributions of Young’s modulus for individual tissues (Panel b). In the case of tissues inflamed P3 and G3, higher stiffness was observed than in tissue samples from other patients. This is also confirmed by Panel c with the average values of elastic moduli. Acute appendicitis was diagnosed in all patients; however, individual appendix tissues were at different stages of inflammation, resulting in variations in tissue structure and differing surface mechanical properties in micro-areas. This could have caused differences in the values of Young’s moduli measured in the study. Differences in these values influenced the standard deviations visible in Panel d. The average value of Young’s modulus and standard deviation equals 496 ± 26 Pa calculated for control appendix tissues, 206 ± 232 Pa for tissues with confirmed purulent inflammation and 114 ± 151 Pa for tissues with confirmed gangrenous inflammation. Structural changes caused by inflammation significantly reduced stiffness.

### 3.2. Shear Rheometry

The macro-rheological properties of human appendix tissues were investigated using a shear rheometer. Tissue samples obtained after surgical appendectomy were subjected to oscillatory shear deformation under various loading conditions. Specifically, a consistent oscillatory shear strain at a fixed frequency was applied while simultaneously compressing the tissue along its axial direction. The degree of compression was systematically varied from 0% to 40% of the initial sample height to assess the impact of compressive strain on the tissue’s shear response. Additionally, to evaluate the nonlinear viscoelastic behavior, oscillatory shear strain with increasing amplitude was applied at a constant frequency, both with and without uniaxial compression. The elastic (storage) modulus (G’) and the viscous (loss) modulus (G”) served as the primary measures of the tissue’s rheological response and were analyzed as a function of shear strain under all loading conditions. Figure 3 displays the storage moduli obtained for individual tissue samples (Panel a) and the corresponding average values (Panel b) for tissue samples subjected to shear loading without compression. Based on the collected results, it can be concluded that, similarly to AFM studies, at the macroscopic level, control appendix tissues are stiffer compared to inflamed ones. The control tissue samples had an average storage modulus of 1136 ± 348 Pa. Samples of appendices histologically assessed as purulent inflammation are less stiff. In this case, the storage modulus was 666 ± 102 Pa. The lowest average value of the storage modulus was obtained for appendix tissues with gangrenous inflammation. It amounted to 642 ± 246 Pa. The difference in stiffness of tissue samples with two types of filling is insignificant. Figure 3 Panel c and Panel d show the normalized values of storage moduli (Panel c) and loss moduli (Panel d) for control and inflamed appendix tissues at different compression ranges (0–40%). In the case of all investigated tissues, compression of as little as 20% causes an increase in the storage modulus. However, this increase is more visible for inflamed tissues.

**Figure 4:**
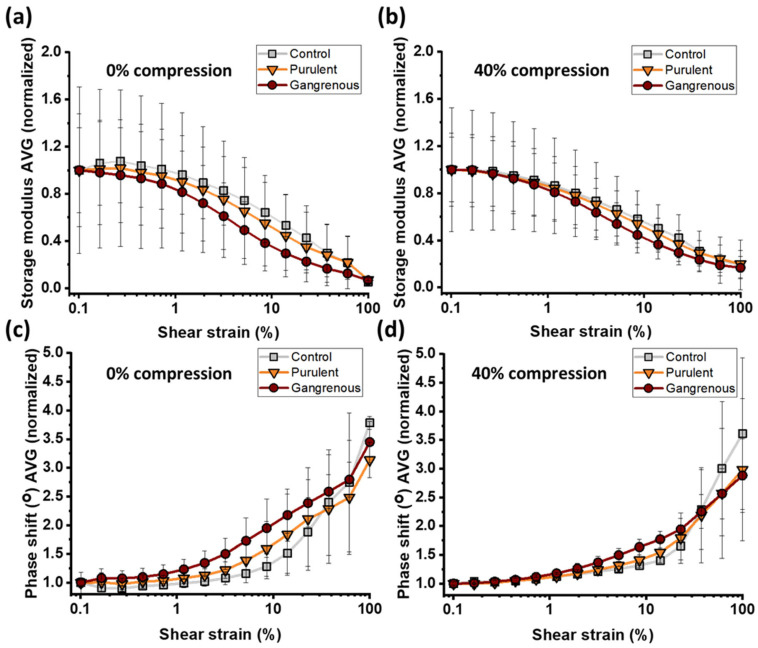
Rheological properties of the control and inflamed vermiform appendix: (**a**) The normalized storage modulus (G’) of control and inflamed tissues as a function of shear strain. (**b**) The normalized storage modulus of control and inflamed tissues as a function of shear strain for 40% of axial compression. The decrease in the G’ with increasing shear strain depicts strain-weakening and is greater for inflamed tissues in a non-compression state. (**c**) The normalized angle of the phase shift as a function of shear strain for control and inflamed tissues without compression. (**d**) The normalized angle of the phase shift as a function of shear strain for the study tissues for axial compression. For 8.5–20% of shear strain, an increased share of viscous properties of inflamed tissue is observed under the influence of increasing shear forces compared to control tissues. An increased share of viscous properties of control tissues is observed for 60% of shear strain.

In the case of 40% compression, the storage modulus of control tissue samples increased by 200%. The appendix tissues with gangrenous inflammation were characterized by the highest increases in storage modulus in compression. Under compression, the storage modulus increased by almost 400% compared to uncompressed tissues. This increase was greater in the case of tissues with purulent inflammation, where the elasticity of the tissues under pressure increased by 350%. Increases in the compression loss modulus are also visible (Panel d). A higher increase was observed for inflamed appendix tissues, as in the case of storage moduli. The results of the dependence of tissue storage moduli as a function of shear strain (Figure 4a for 0% compression and Figure 4b for 40% compression) and phase shift angles (Figure 4c for 0% compression and Figure 4d for 40% compression) show differences in the behavior of control and inflamed appendix tissue, but the differences are not as clear as in the case of the stiffening phenomenon in compression.

In the absence of axial compression and increasing shear strain, tissue samples show a decrease in elastic modulus (Panel a). In the case of a shear strain of 10%, differences in the mechanical behavior of the appendix tissues are visible. The inflamed appendix tissues weaken more than the control ones. This effect is less visible in axial compression (Panel b). In the case of tissues without compression, for a shear strain of 8.5%, a greater increase in the phase shift angle is visible for samples in the inflamed state (Panel c). This angle was 26° in the case of purulent tissues and 30° in the case of gangrenous tissues. Control tissue had an average phase shift angle of 22°. The increase in the angle means that pathological tissues are more dissipative when the angular strain increases to 30%. This property does not intensify during compression.

## 4. Discussion

In recent years, the mechanical properties of different human tissues have been determined, but changes in their properties during disease states, for example, inflammation or cancer, have not been fully understood. We have recognized the specific properties of cancerous tissues [14,15], while inflammatory tissues are less known [6,17]. This study examined the use of a shear rheometer and an atomic force microscope to measure the rheological properties of inflamed appendix tissues. We aimed to identify mechanomarkers of inflammation that could enhance existing diagnostic methods and help differentiate inflammation from the development of cancer.

In the first stage, tissue stiffness was measured using an AFM microscope equipped with a spherical probe. The results of measurements of the modulus of elasticity—Young’s modulus of appendix tissues indicate a similar range of values as in the case of the data obtained from the tissues of the intestine [14], stomach [17], or in the list of properties of various types of tissues contained in the study [10]. Based on the results, it can be directly concluded that the inflamed appendix tissues are less stiff than the control tissues. The Young’s moduli of inflamed appendix tissues, calculated based on force curves from the AFM microscope, are lower than those of control tissues. This is the opposite of carcinogenesis processes, in the example of colorectal cancer, where a marked increase in the stiffness of cancer tissues [14], compared to healthy tissues, is associated with overexpression of extracellular matrix proteins, increased matrix fibrosis and cross-linking during tumor progression [14,22,34,35,36,37]. On the other hand, inflammatory processes generate changes in the mechanical properties of tissues, inducing immune cell infiltration (neutrophils) in the appendiceal mucosa, submucosa, and muscularis propria, serosal exudate [38,39,40,41,42,43], which results in a decrease in the mechanical integrity of the tissues. These are not processes that strengthen the specific protein skeleton of the extracellular matrix, which is a scaffold that transfers mechanical loads [44,45,46]. The fibers of this matrix carry loads in compression and torsion, especially if there are rigid particles between them and interactions between them [46]. Additionally, inflammation can lead to impaired cellular functions in maintaining tissue homeostasis. At the cellular level, it causes disruptions in cytoskeletal structure (polymer structures, which control the shape of eukaryotic cells and provide a basis for movement and cell division) and alters the expression of water and ion channels, and this kind of imbalanced anabolic-catabolic response to inflammation leads to the breakdown of the ECM [47].

The effect of reducing the stiffness of inflamed tissues compared to healthy tissues was also observed in our previous study [17], where the nanomechanical characteristics of *Helicobacter pylori* infection were investigated. The decreased stiffness of the infected gastric mucosa was associated with the deregulation of cellular functions caused by specific *Helicobacter pylori* virulence factors. Clear differences in stiffness between the control and inflamed tissues indicate that stiffness may be a marker enabling the differentiation of healthy and inflamed vermiform appendix. Differences in the stiffness between tissues with confirmed necrotic processes and those with purulent inflammation suggest that the presence of tissue necrosis and the associated tissue destructions may further reduce tissue stiffness. Nevertheless, these differences are not significant. In both cases, the primary cause of decreased elasticity is cell infiltration and the weakening of the extracellular matrix. To define a mechanical profile of vermiform appendix tissues that can distinguish between healthy and inflamed tissues, particularly to differentiate the etiology of inflammation, a larger sample group would need to be examined. However, the results of this study confirm the feasibility of measuring the mechanical properties of appendix tissues using AFM. The stiffness profiles observed in our study for control tissue are characterized by a relatively wide distribution of Young’s modulus, indicating its mechanical heterogeneity, while the stiffness profiles of inflamed appendix tissues are characterized by sharper peaks, similar to the study [17]. In the case of cancer tissues [14], the range of Young’s modulus was significantly extended, which resulted in the shape and shift of histograms towards higher values of Young’s modulus. Differences in the Young’s modulus of inflamed appendix tissues may result from varying degrees of local tissue inflammation. For two samples of inflamed tissue, stiffness was found to be higher than in the other samples of the group. This may be attributed to the specific nature of measurements made using the AFM nanometric tip. Inflammation is a progressive process that alters the internal structure of tissues. The examined appendices were at different stages of inflammation, with varying levels of cellular infiltration influencing their mechanical properties. The two samples mentioned may have been at an earlier stage of inflammation, where their internal structure, and thus their mechanical properties, had not yet been significantly altered. AFM measurements are performed only on the surface layer of the tissue in selected micro-areas. Due to the small contact area of the AFM tip and its relatively shallow indentation, the resulting deformations are minimal, affecting only single cells or specific cellular structures. The issues with conducting such measurements are described in the study [31]. Tissues are complex 3D structures. A large number of force maps must be analyzed to determine the mechanical properties of tissues using an AFM microscope. It may be more appropriate to perform bulk rheological measurements on tissue samples.

Large biopsy samples permit the analysis of macrorheological properties via shear rheometry, enabling the characterization of tissue behavior under complex deformation states involving simultaneous shearing and compression. This approach facilitates the determination of storage modulus (G’) and loss modulus (G”), providing insights into the dynamic response of tissues under various shear conditions, ultimately furthering our understanding of their mechanics in physiological settings. Similarly to the AFM, the rheometry studies indicated that inflamed appendix tissues are clearly less stiff than healthy tissues, which can be observed as a decrease in the storage modulus of inflamed tissues compared to the control ones. The decrease in storage modulus in inflamed appendix tissues is the opposite trend of tissues undergoing carcinogenesis. The causes of such mechanical changes regarding Young’s moduli of inflamed tissues were described here in the section. Therefore, we postulate that the storage modulus G’, similar to Young’s modulus, may be a promising mechanomarker of vermiform appendix inflammation.

The results of tissue elasticity measurements during axial compression revealed the property of all control and inflamed appendix tissues to stiffen in compression. This stiffening effect is in agreement with the results previously published. The strengthening of tissue during compression was described earlier as the compression stiffening effect [8,44,45,46,48]. Colon cancer tissues reacted to compression by increasing elasticity to a greater extent than healthy tissue [14]. Here, the stiffening effect is greater for inflamed appendix tissues than for control ones. In the case of colon cancer tissues [14], this increase for an axial deformation of 40% was clearly greater than in the case of inflamed appendix tissues. In summary, inflamed appendix tissues are less stiff than healthy ones, but they also exhibit a stiffening effect under compression, similar to cancerous tissues. This may be related to the nature of internal tissue changes resulting from the development of the disease. Tissue rheology results strictly from the interaction between the polymer network and the cells retaining volume in the network [45,46]. A previous study [46] describes a tissue model that explains the specific stiffening properties under compression. The tissue is characterized as a compressible phase of the extracellular matrix and incompressible cellular elements. During compression, this arrangement causes fluid to flow out of the matrix, and contact between the cellular elements generates mechanical resistance. In cancerous tissues, in addition to an increased number of pathological cells, there is also a strengthening of the extracellular matrix. In contrast, in inflammatory tissues, immune cell infiltration plays the main role. In compression, cells induce deformation in the surrounding matrix. If the matrix is stronger, as seen in cancerous tissues, it resists deformation more effectively, generating greater feedback forces [45]. In the case of inflammation, increased packing of elastic cells within the matrix can intensify the compression-stiffening effect compared to that in control tissues. Consequently, during compression, there is a greater proportion of interactions among the elastic cells themselves when they are in close proximity. The results do not indicate any clear differences in the storage modulus or compression behavior between tissue samples with purulent inflammation and those with visible necrosis. Mechanistically, cell infiltration occurring in both types of inflammation plays the most important role. We can conclude that the changes observed in the mechanical response of inflamed vermiform appendix to loads may enable the identification of specific mechanomarkers of inflammation—in particular stiffness, quantified by the storage modulus G’, as well as increased levels of compression stiffening.

Another phenomenon observed when increasing the amplitude of shear strains is a decrease in the elastic modulus of tissues. This phenomenon was also observed in the studies [14,46] and is characteristic of sheared tissue structures. Shearing leads to the breaking of connections between the cells and the matrix, resulting in a weakening of the structure [46]. Inflamed appendix tissues, especially those with confirmed necrosis, weaken more than control tissues with increasing strain amplitude. Necrotic tissues have a higher degree of mechanical degradation of the structure. However, the effect of the decrease in the value of tissue elastic modulus with increasing shear stress is not as pronounced as in the case of cancer tissues [14], where increased collagen expression and cross-linking strengthened more cancerous tissues and where increased shearing causes breaking of connections between elastic cells and the matrix resulting in a weakening of the tissue.

Changes in the phase shift angle with increasing strain amplitude indicate greater energy dissipation of inflamed tissues, especially gangrenous inflammation, compared to healthy tissues. The structure of inflamed tissues has more fluid characteristics due to serosal exudate, cell infiltration, presence of necrosis and degradation of the ECM, which weakens the tissue structures. High levels of shear weakening or energy dissipation can be proposed as another potential mechanomarker of tissue inflammation. Attempts to study tissues using magnetic resonance elastography (MRE) suggest a high potential of the dissipative feature of tissue rheology as a new marker in their pathology [22,49,50,51,52]. However, the group of tissues examined should be increased. In the case of research on a large group of patients, it is possible to create a mechanical profile of inflamed tissues, even with a separation into different etiologies of the disease. In our experiments, tissue samples were frozen before testing. We extend the list of biological materials exhibiting compressional stiffening and shear weakening. We cannot determine precisely the mechanical parameters of vermiform appendices, but we are able to compare the mechanical properties of control and inflamed tissues. The significant value of this work is the fact that biopsies were taken from children with no signs of other inflammatory conditions, including chronic ones. The children also did not have any diets or habits that could affect the condition and mechanical properties of their internal organs. The differences in the observed stiffness of inflamed tissues compared to the healthy ones are pronounced. Due to specific structural changes, inflamed tissues also respond differently than healthy and cancerous tissues under axial and shear deformations [14]. By understanding the observed differences in mechanical properties between healthy and inflamed tissues, we can distinguish them through rheological measurements. Gaining insights into the mechanical properties of purulent and gangrenous inflamed appendices could aid in identifying the inflammation’s etiology and evaluating its severity. Miniaturization and simplification of rheological techniques could allow measurements to be taken even when only a small volume of tissue is available [53].

Given that the molecular processes in inflammatory and cancerous tissue development differ, resulting in distinct mechanical changes, rheological measurements and mechanical profiles of tissues could enable the diagnosis of pathological changes, including the early detection of cancerous transformations, potentially earlier than standard histopathological examinations. Mechanomarkers obtained during rheological tests, such as Young’s modulus or storage modulus, can enhance standard histopathological procedures, enabling us to distinguish the tissue processes of carcinogenesis from inflammation. This can lead to a better understanding of the relationship between pathological tissue states and their mechanical properties and support the development of new diagnostic methods.

Future studies should continue assessing rheological properties using atomic force microscopy and shear rheology methods for other diseased tissues exhibiting chronic inflammatory states, which are often linked to impaired regeneration and tumor development (e.g., chronic gastric mucosal inflammation due to *Helicobacter pylori* infection and the associated tumor development in this organ). Continuing this research, with data on how mechanical properties change in inflammatory states, will enable the exploration of potential new signaling pathways that influence tissue mechanical properties and facilitate the creation of a theoretical model describing tissue behavior under axial and shear forces. Such studies would also highlight the correlation between changes in mechanical properties and alterations in the extracellular matrix structure, which is largely responsible for the mechanical behavior of biological tissues and is a focus of numerous studies [22,24,44,46,48]. Expanding comprehensive rheological studies to include a broad range of human tissues affected by inflammatory processes, increasing the sample size alongside models that mimic the extracellular matrix, and exploring new signaling pathways affecting mechanical properties may lead to the development of innovative diagnostic and therapeutic methods, thereby improving patient prognosis.

## 5. Conclusions

Based on the results, it can be concluded that atomic force microscopy can be a method for measuring mechanomarkers of pathological tissue states. However, this method has significant drawbacks. It is time-consuming and gives us a mechanical image of the tissue only from its surface. Shear rheology methods give us a mechanical view of the entire tested sample, which is very important because solid tissue sections are complex 3D structures where molecular, cellular and architectural changes manifest themselves in diverse mechanical properties in micro-ranges. A comparison of the mechanical properties of the healthy and inflamed appendix tissues indicates that the pathological appendix tissues are softer than the healthy ones. Additionally, inflamed appendix tissues exhibit a greater compression stiffening effect and shear weakening compared to healthy tissues.

If we examine a large group of patients using AFM or shear rheology methods, obtaining mechanical profiles of healthy and diseased tissues, the specific mechanical properties in inflammation mentioned in the study might be better defined as new mechanomarkers. Mechanomarkers like Young’s modulus or storage modulus G’ can enhance histopathological methods that differentiate the tissue process of carcinogenesis and inflammation. This will enable us to better understand the correlation between the disease state of tissues and their mechanics, especially if methods of measuring the mechanical properties of human tissues intraoperatively or non-invasively will be improved in the near future.

## Data Availability

The original contributions presented in the study are included in the article, further inquiries can be directed to the corresponding author.
